# A Highly Selective and Sensitive Fluorescent Chemosensor for Detecting Al^3+^ Ion in Aqueous Solution and Plant Systems

**DOI:** 10.3390/s19030623

**Published:** 2019-02-01

**Authors:** Chia-Lin Li, Ping-Hsuan Lu, Shih-Feng Fu, An-Tai Wu

**Affiliations:** 1 Department of Chemistry, National Changhua University of Education, Changhua 50058, Taiwan; cindy88034@gmail.com (C.-L.L.); kenlupr@gmail.com (P.-H.L.); 2 Department of Biology, National Changhua University of Education, Changhua 50058, Taiwan

**Keywords:** aluminum, rice seedlings, fluorescence

## Abstract

The solubilized form of aluminum, Al^3+^, is present under acid soil conditions and toxic to both animals and plants. Detecting and quantifying Al^3+^ is vital for both chemistry and biology. A new Schiff-based fluorescent turn-on sensor (probe **L**) for the selective detection of the Al^3+^ ion was synthesized by coupling 2-hydroxy-1-naphthaldehyde and 2-aminoisoindoline-1,3-dione, and the structure was characterized by nuclear magnetic resonance spectra. The probe **L** exhibited an excellent selective and sensitive response to the Al^3+^ ion over other metal ions in DMSO-H_2_O (1:9 *v*/*v*). Fluorescence quantification revealed that probe **L** was promising for the detection and accumulation of Al^3+^. Treating rice seedlings with Al^3+^ at 25–200 μM inhibited their growth. Al^3+^ treatment produced reactive oxygen species in rice roots. Practical applications of the fluorescent probe for the quantification of Al^3+^ in water samples and rice seedlings are demonstrated. Detecting the Al^3+^ ion with the probe **L** is easy and a potential alternative to existing analytical methods. The method can be used for detecting the Al^3+^ content of aqueous solution and plant systems. The novel fluorescent probe **L** has good potential for monitoring Al^3+^ content in the environment and biological systems.

## 1. Introduction

Aluminum (Al) is the third most abundant element in the earth’s crust. With acidic rain and industrial pollution, a trivalent cation of Al^3+^ is solubilized and widely exists in the environment. Approximately 40 to 50% of the world’s arable soils are acidic, and the toxicity of Al is considered an important limiting factor of plant productivity on acidic soils [1,2,3]. Excessive Al content in contaminated soil leads to a decrease in soil microbial content and hampers plant growth [4]. Additionally, Al is widespread in food additives, pharmaceuticals, and storage/cooking utensils. Al^3+^ ions, the solubilized form of Al, enhance the peroxidation of phospholipids and proteins in cell membranes. Al^3+^ ions can move in the human body via food and water sources. Heavy metal intake by human populations through the food chain damages the nervous system and can induce Alzheimers and Parkinsons disease and amyotrophic lateral sclerosis [5,6,7,8].

Al in its ionic form, Al^3+^, is found in most animal and plant tissues and in natural water. Detecting and quantifying Al^3+^ is crucial for controlling its concentration in the biosphere and its direct impact on human health. Various kinds of sensors have been reported for selective Al^3+^ detection [9,10,11,12,13,14,15,16,17,18,19,20,21,22,23,24,25,26]. However, these sensors are limited by their complicated synthesis, low water solubility, and lack of sensitivity. For practical applications, a water-soluble sensor for detecting Al^3+^ is still in great demand. Recently, several fluorescent probes have been synthesized for sensing Al^3+^ in cells [27,28,29,30]. The use of fluorescent probes for biosensing and bioimaging the ion in animal cells has also been reported. However, these fluorescent probes have not yet been examined for their application in plant systems. The detection of metal ion by fluorescent probes in plant tissues has always been problematic because of interference from the intrinsic fluorescence of the cell wall and secondary metabolites such as tannins, alkaloids, and polyphenols [31,32].

**Scheme 1:**
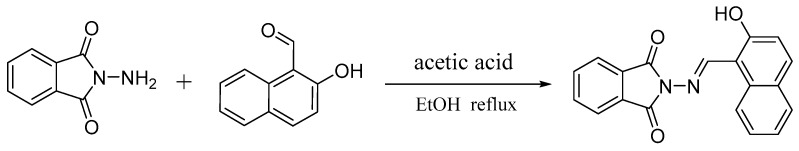
Synthesis of probe **L**.

In this study, we designed a fluorescent sensor, probe **L** (Scheme 1), that showed a selective colorimetric change toward Al^3+^ among the other tested metal ions. To demonstrate the potential of probe **L** for biological detection, we first analyzed the effect of Al^3+^ exposure on root elongation in rice (*Oryza sativa* L. cv. TN67). The toxicity and accumulation of Al^3+^ were evaluated in rice seedlings and we used probe **L** to quantify the Al^3+^ level in rice seedlings. The toxicity and accumulation of Al^3+^ inhibited cell elongation and cell division, which led to the stunted growth of roots. Al^3+^ treatment may decrease the uptake of water and nutrients and negatively affect plant growth.

## 2. Materials and Methods

### 2.1. Synthesis of Probe L

The synthesis of probe **L** was based on the following method (Scheme 1). 2-hydroxy-1-naphthaldehyde (0.278 g, 1.61 mmol) was added to a solution of 2-aminoisoindoline-1,3-dione (0.303 g, 1.87 mmol) in dry ethanol (15 mL) with two drops of acetic acid. The reaction mixture was refluxed overnight and then the solvent was removed under reduced pressure. The resulting solid was washed with cooled EtOH repeatedly to give probe **L** (0.469 g, 80%) as a yellow solid; melting point: 234.0 °C; ^1^H NMR (300 MHz, DMSO-*d*_6_) δ 11.95 (s, 1H), 10.07 (s, 1H), 8.62 (d, 1H, *J* = 4.4 Hz), 8.05 (d, 1H, *J* = 4.4 Hz), 7.99–7.90 (m, 5H), 7.66–7.61 (m, 1H), 7.47–7.42 (m, 1H), 7.28 (d, 1H, *J* = 4.5 Hz); ^13^C NMR (75 MHz, DMSO-*d*_6_) δ 164.33, 159.50, 159.26, 134.99, 131.77, 130.16, 129.04, 128.44, 127.99,123.94, 123.54, 122.28, 118.67, 108.61; HRMS(EI): calculated for C_19_H_12_N_2_O_3_(M + H), *m*/*z* 316.0848; resulting *m*/*z* 316.0839. (Figures S1–S3).

### 2.2. Plant Materials and Growth Conditions

Rice plants (*Oryza sativa* L. cv. TN67) were grown as described in [33] with minor modification. The seeds were incubated at 37 °C for 3 d in darkness. Ten germinated seeds with coleoptiles reaching 2 mm in length were transferred to a Petri dish (9 cm in diameter) with a filter paper. The rice seedlings were incubated with deionized water supplemented with Al(ClO_4_)_3_ (Sigma-Aldrich, St. Louis, MO, USA) from 25 to 200 μM, treated with Al^3+^ solution (10 mL in each Petri dish) for 10 d, then subcultured with fresh solution for another 10 d. Control seedlings were incubated without Al^3+^. The rice seedlings were analyzed for root elongation and reactive oxygen species (ROS) production. For measuring dry weight and accumulation of Al^3+^, samples were dried by incubation at 37 °C for 7 d. Data were obtained from at least three biological replicates.

### 2.3. Use of Probe L to Determine Al Concentration in Rice

Roots of 20-day-old rice seedlings were rinsed thoroughly with deionized water and oven-dried for 2 d at 37 °C. Ten rice seedlings were ground into fine powder and dissolved with 24× deionized water. The extract was mixed with an equal volume of chloroform and then centrifuged at 2000 g for 2 min. Al^3+^ content was determined in the supernatant (270 μL) by supplementation with the fluorescence probe **L** (30 μL) dissolved in 10% DMSO. Samples of 400 μM probe **L** were incubated at 2 °C for 20 min. The Al^3+^-induced specific fluorescence was determined by fluorescence spectrometry (FP-8300, JASCO, Germany) at 375 nm excitation. Data are mean values from three independent biological replicates.

### 2.4. In Situ Detection of Reactive Oxygen Species (ROS)

Rice roots were treated with various concentrations of Al(ClO_4_)_3_ for 14 d. H_2_O_2_ levels were detected by treating roots with 3,3’-diamninobenzidine (DAB). To detect H_2_O_2_, root samples were placed in a solution [10 mM 2-(N-morpholino)ethanesulfonic acid (MES) buffer, pH 3.8] of 1 mg mL^−1^ DAB overnight in darkness. Root samples were rinsed briefly with deionized water. Staining was observed by stereomicroscopy.

## 3. Results and Discussion

### 3.1. Absorption and Fluorescence Studies of Probe L Toward Various Metal Ions

**Figure 1:**
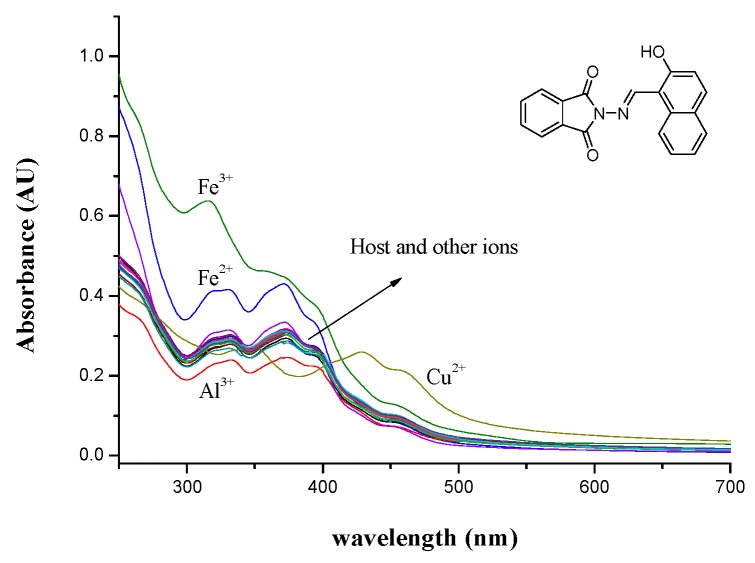
UV/vis spectra for probe **L** (40 μM) in DMSO-H_2_O (1:9 *v*/*v*) with the addition of 5 equiv. of various metal ions.

**Figure 2:**
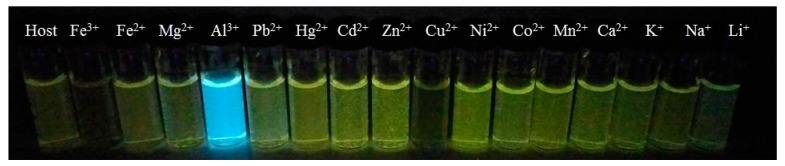
Color changes by UV light for probe **L** (40 μM) with the addition of 5 equiv. of various metal ions.

**Figure 3:**
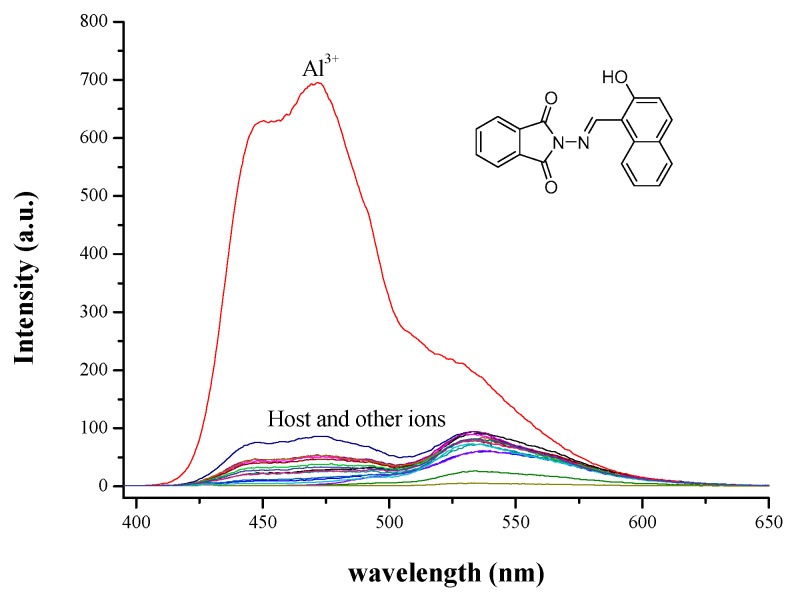
Fluorescence emission spectra for probe **L** (40 μΜ) in the presence of 5 equiv. of various metal ions in DMSO-H_2_O (1:9 *v*/*v*).

We used by UV/vis and fluorescence measurements to investigate the chemosensor behavior of probe **L** with the following 16 metal ions (as perchlorate salts): Li^+^, Na^+^, K^+^, Ca^2+^, Mg^2+^, Mn^2+^, Hg^2+^, Fe^2+^, Fe^3+^, Co^2+^, Ni^2+^, Cu^2+^, Pb^2+^, Cd^2+^, Zn^2+^, and Al^3+^ in DMSO-H_2_O (1:9 *v*/*v*). The solution of probe **L** itself showed two absorption bands at 320 nm and 370 nm, respectively (Figure 1). The addition of Fe^3+^ and Fe^2+^ to the solution enhanced the absorption band. In the presence of Cu^2+^, the absorption spectrum of probe **L** was red-shifted, accompanied by a striking color change from dark green to black, which could be seen under UV light (Figure 2). In the presence of Al^3+^, the intensity of the absorption spectrum of probe **L** was decreased, accompanied by a striking color change from dark green to blue, which could be detected under UV light (Figure 2). These changes in absorption bands indicated a partial interaction between the ions and probe **L**. As compared with the weak emission band of probe **L** alone, the addition of Al^3+^ generated an additional fluorescent-enhanced emission band at 472 nm (Figure 3). The enhanced efficiency at 472 nm was 11-fold greater than the control without Al^3+^ (Figure S4). The fluorescent enhancement may be attributed to the formation of a rigid system after binding with Al^3+^.

### 3.2. Fluorescence Titration and Binding Studies

**Figure 4:**
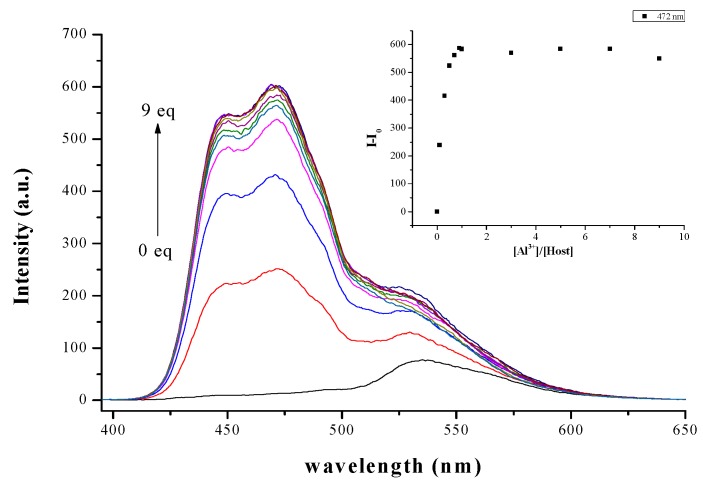
Fluorescence emission spectra for probe **L** (40 μM) in DMSO-H_2_O (1:9 *v*/*v*) with increasing concentrations of Al^3+^. Inset: intensity (at 540 nm) for probe **L** as a function of Al^3+^ equiv.

To further investigate the chemosensing properties of probe **L**, probe **L** was fluorescence-titrated with Al^3+^. The changes in fluorescence spectra for probe **L** as a function of Al^3+^ content are shown in Figure 4. With increasing amounts of Al^3+^ added to a solution of probe **L** in DMSO-H_2_O (1:9 *v*/*v*), the emission band at 530 nm gradually increased, and concomitantly a new emission band appeared that peaked at 472 nm. A Job plot indicated a 1:1 stoichiometric complexation of probe **L** with Al^3+^ (Figure S5). Additionally, the formation of a 1:1 complex between probe **L** and Al^3+^ was confirmed by the appearance of a peak at *m*/*z* 393, assignable to [probe **L** + Al^3+^ + 3H_2_O − 4H^+^] in the ESI/MS (Figure S6). From the fluorescent titration profiles, the association constant for probe **L**-Al^3+^ in DMSO-H_2_O (1:9 *v*/*v*) was determined as 3 × 10^7^ M^−1^ by a Hill plot (Figure S7). By using the abovementioned fluorescence titration results, the detection limit for Al^3+^ was estimated as 1 ppb.

### 3.3. Application in Water

In order to determine the optimal pH condition of the probe **L** in the water, we examined the effect of pH on the emission bands of the probe **L**–Al^3+^ complex in DMSO-H_2_O (1:9 *v*/*v*) solution. The fluorescence intensity showed a significant emission band (Figure S8) accompanied by a marked color change from dark green to light blue (Figure S9), which could be detected under UV light in the pH range 5–10. Thus, probe **L** could be applied for the analysis of environmental aqueous samples with a relatively wide pH range.

**Figure 5:**
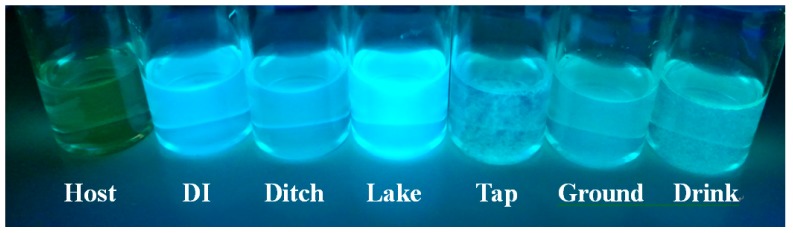
Color changes by UV light for probe **L** (40 μM) in different sources of water (DMSO : H_2_O = 1 : 9 *v*/*v*) containing 5 equiv. of Al^3+^.

The practical application of probe **L** (40 μM) for selective sensing of Al^3+^ (5 equiv.) in different sources of water (DMSO: H_2_O = 1 : 9 *v*/*v*) was demonstrated (Figure 5). Al(ClO_4_)_3_ was first dissolved in water of the above source, followed by the addition of probe **L** to each water sample. All water samples containing Al^3+^ showed a clear color change from dark green to light blue. The result indicated that probe **L** can detect Al^3+^ in different water sources.

**Figure 6:**
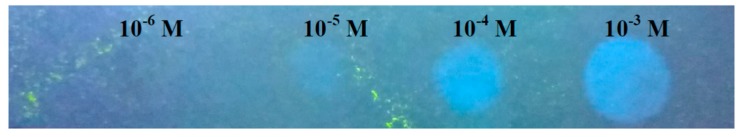
The color changes of probe **L** observed by UV light upon the addition of Al^3+^ at different concentrations in the filter paper.

Testing paper detection is an inexpensive approach to detect the presence of an analyte. Toward this goal, we first coated filter paper with probe **L** by soaking in a DMSO-H_2_O (1:9 *v*/*v*) solution followed by air drying. Al^3+^ at different concentrations was added to this probe **L**-coated test paper: the color of the paper changed from dark green to light blue instantly (Figure 6), caused by the interaction of probe **L** and Al^3+^, therefore implying that the test paper specifically recognized Al^3+^. The detection limit of Al^3+^ with this probe **L**-coated test paper was 1.0 × 10^−5^ M.

### 3.4. Effect of Al^3+^ Stress on Root Elongation and Oxidative Burst in Rice

**Figure 7:**
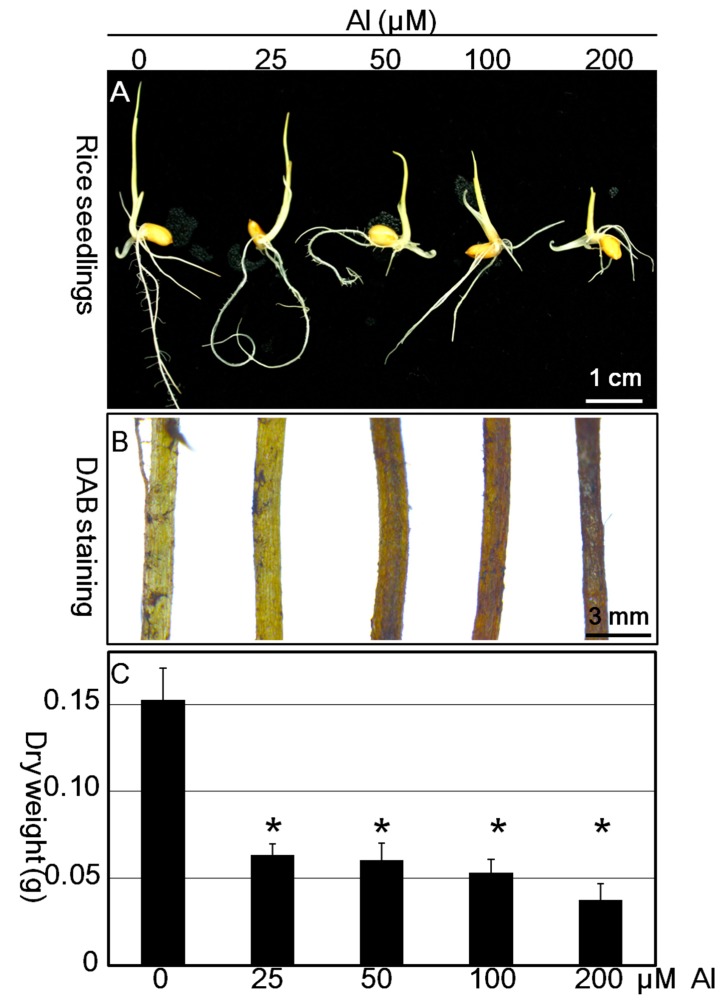
**Growth and production of reactive oxygen species (ROS) in rice seedlings with Al treatment.** (**A**) Rice seedlings were treated with different concentrations of Al(ClO_4_)_3_ (0, 25, 50, 100, and 200 μM) for 20 d, and the morphology of rice seedlings was photographed. (**B**) Al-induced ROS accumulation in rice roots. Root samples treated with Al^3+^ were stained with 3, 3’-diamninobenzidine (DAB) for determining H_2_O_2_. (**C**) Dry weight of rice seedlings with treatment of Al. Data represent the mean ± SD of three independent experiments. Results are the mean ± SE (n = 10). * Significantly different from the control at P < 0.05 by paired *t* test.

**Figure 8:**
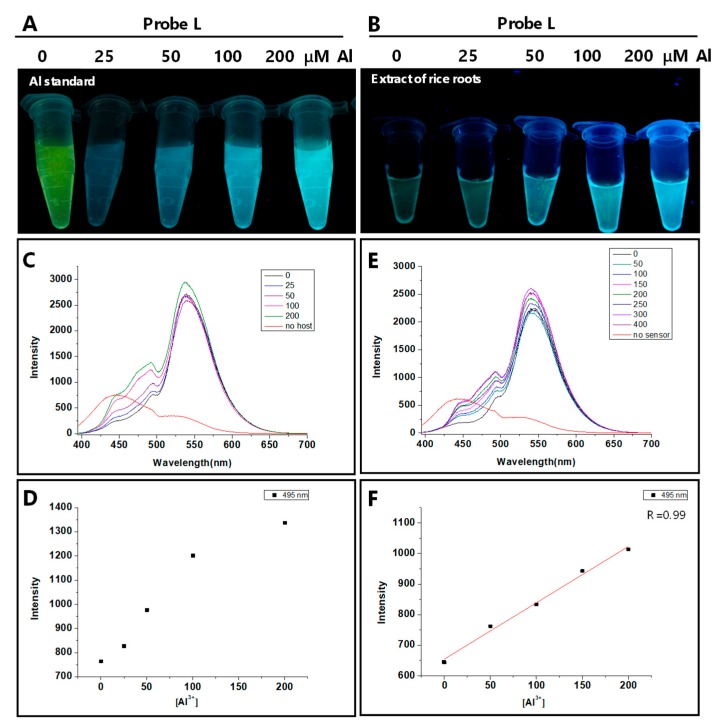
**Quantitative determination of Al concentrations in rice seedlings.** (**A**) Fluorescence probe **L** with the addition of various concentrations of Al(ClO_4_)_3_ solution (0, 25, 50, 100, and 200 μM) was excited by a handheld UV lamp with 345 nm excitation. The blue emission was photographed immediately in the dark. (**B**) Fluorescence probe **L** with the addition of rice extracts on treatment with various concentrations of Al^3+^. The samples were excited by UV, and blue emission was photographed. (**C**) Measurement of fluorescence intensity of probe **L** at 495 nm with the addition of rice extracts on treatment with various concentrations of Al^3+^. All data were measured after 20 min nm. (**D**) Fluorescence values corresponding to each treatment in (C) were plotted. (**E**) Measurement of fluorescence intensity of probe **L** with increasing concentrations of Al^3+^ standards (0, 50, 100, 150, 200, and 300 μM) dissolved in control seedling extracts. (**F**) A linear calibration curve was constructed on the basis of fluorescence intensity and Al^3+^ standards. Three replicates were performed with nearly identical results.

The inhibition of root growth is the primary response of the plant exposed to heavy metals. We analyzed the effect of Al^3+^ exposure on root elongation to evaluate Al^3+^ toxicity in rice seedlings. After exposing rice seedlings to Al^3+^ for 14 d, root elongation was measured. At 25 and 50 μM, Al^3+^ significantly reduced root elongation as compared with control conditions (Figure 7A). The root elongation decreased with increasing Al^3+^ concentration. At 100 μM Al^3+^, root elongation was reduced to about 50% of the control conditions. At 200 μM Al^3+^, root growth was completely inhibited. Al^3+^ at 200 μM inhibited root elongation by approximately 80%. We determined production of ROS such as H_2_O_2_ by treating roots with DAB. Treatment with 25 and 50 μM Al^3+^ induced more ROS production compared with control plants (Figure 7B). The ROS level was greater with 100 and 200 μM Al^3+^ than in controls (Figure 7B). Accordingly, the dry weight of roots was significantly decreased with 100 and 200 μM Al^3+^ (Figure 7C). It was reported that treatment of 200 μM perchlorate had subtle effects on root length and root weight of rice plants [34]. With treatment of 400 μM perchlorate, most of the rice varieties exhibited noticeable toxicity symptoms such as wilting, abscission, and even necrosis [34]. In our study, root weight was markedly inhibited by treatment with 25 μM Al(ClO_4_)_3_ (Figure 7C). It is suggested that Al^3+^ resulted in the heavy metal stress in the rice plants. Thus, Al^3+^-induced ROS production may inhibit growth in rice seedlings (Figure 8B,C). Thereafter, we treated rice seedlings with 25–200 μM Al^3+^ for the quantification of Al^3+^ by the colorimetric fluorescent probe.

### 3.5. Practical Applications of Probe L for Quantifying Al Content in Rice Seedlings

With the excellent response of probe **L** to Al^3+^ in aqueous solution, we investigated its ability to detect Al^3+^ content in rice seedlings. The intensity of blue fluorescence was increased with increasing Al^3+^ solution standard from 25 to 200 μM (Figure 8A). Extracts of Al^3+^-treated rice seedlings were incubated with the fluorescent probe. Extracts with 50 μM Al^3+^ showed mild fluorescence. The maximal fluorescence intensity was observed with 200 μM Al^3+^ (Figure 8B). The intensity of fluorescence was further quantified by fluorescence spectroscopy. Fluorescence emission spectra were measured at 495 nm (Figure 8C). Fluorescence intensity was increased with increasing Al^3+^ concentration from 25 to 100 μM (Figure 8D). The highest levels of Al^3+^ occurred with 200 μM Al^3+^. The stationary phase of Al^3+^ accumulation in rice seedlings may be due to reaching its saturation level (Figure 8D). Therefore, Al^3+^ accumulation in rice was associated with Al^3+^ concentration, with greater accumulation at 200 μM Al^3+^.

An Al^3+^ standard curve was constructed by linear regression with the Al^3+^ calibration standards in control seedling extracts (Figure 8E). The standard curve showed an acceptable regression line with concentration range 0 to 300 μM (Figure 8F). We then determined Al^3+^ content in 25, 50, 100, and 200 μM-treated samples. The level of heavy metals detected in rice seedlings was 98, 200, 353, and 446 μM, respectively. The differences in Al^3+^ toxicity (Figure 7) may be related to the accumulation in rice (Figure 8). This result agrees with the ratiometric fluorescence changes observed in aqueous solutions. Therefore, the fluorescence probe can be used for the quantification of Al^3+^ content in plants.

## 4. Conclusions

We developed a new fluorescent probe, probe **L**, for detecting Al^3+^ content. The probe exhibited rapid, highly selective and sensitive detection of Al^3+^ content, with distinct changes in fluorescent emission. Fluorescent quantification demonstrated that probe **L** could be used to detect plant samples with heavy metal Al^3+^ pollution. This study provides a promising fluorescent probe for the detection and accumulation of Al^3+^ that could be an easy potential alternative to existing analytical methods for investigating Al^3+^ content in plants.

## Supplementary Materials

The following are available online at http://www.mdpi.com/1424-8220/19/3/623/s1, Figure S1: ^1^H NMR (DMSO-*d*_6_) spectra for probe **L**, Figure S2: ^13^C NMR (DMSO-*d*_6_) spectra for probe **L**, Figure S3: High-resolution mass spectrometry data for probe **L**, Figure S4: Fluorescence intensity at 536 nm (λ_ex._ = 320 nm) for probe **L** (40 μM) in the presence of 5 equiv. of various cations in DMSO-H2O (1:9 *v*/*v*), Figure S5: Job plot of probe **L** and Al^3+^, Figure S6: ESI mass spectra [(probe **L** + Al^3+^ + DMSO + ClO4^−^) + 2Na^+^], Figure S7: Hill plot, Figure S8: Fluorescence emission intensity for probe **L** (40 λM) in the presence and absence of Al^3+^ (5 equiv) at different pH, Figure S9: Color changes by UV light for probe **L** with the addition of 5 equiv. of Al^3+^ at different pH value.
