# Supplementary material for: A Highly Selective and Sensitive Fluorescent Chemosensor for Detecting Al3+ Ion in Aqueous Solution and Plant Systems
Source: Sensors (Basel). 2019 Feb 1;19(3):623. doi: 10.3390/s19030623 (PMC6387123; doi:10.3390/s19030623)
Supplement: Supplementary file 1 [file sensors-19-00623-s001.pdf]

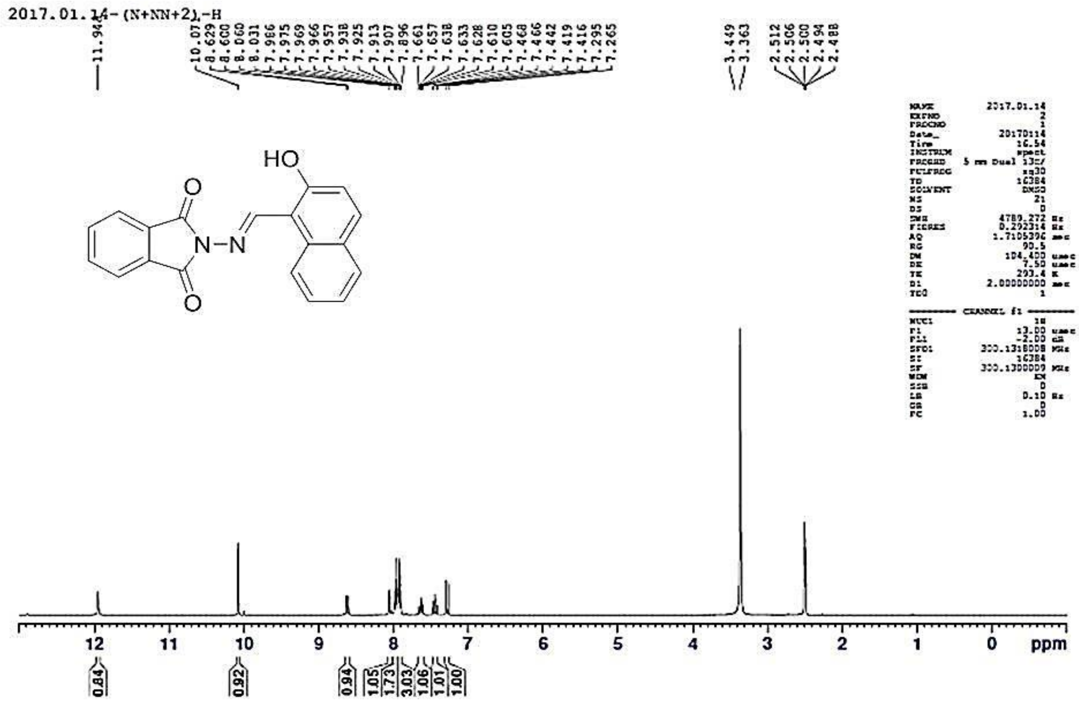

Fig. S1. ^{1}H NMR (DMSO-d_6) spectra for probe **L**

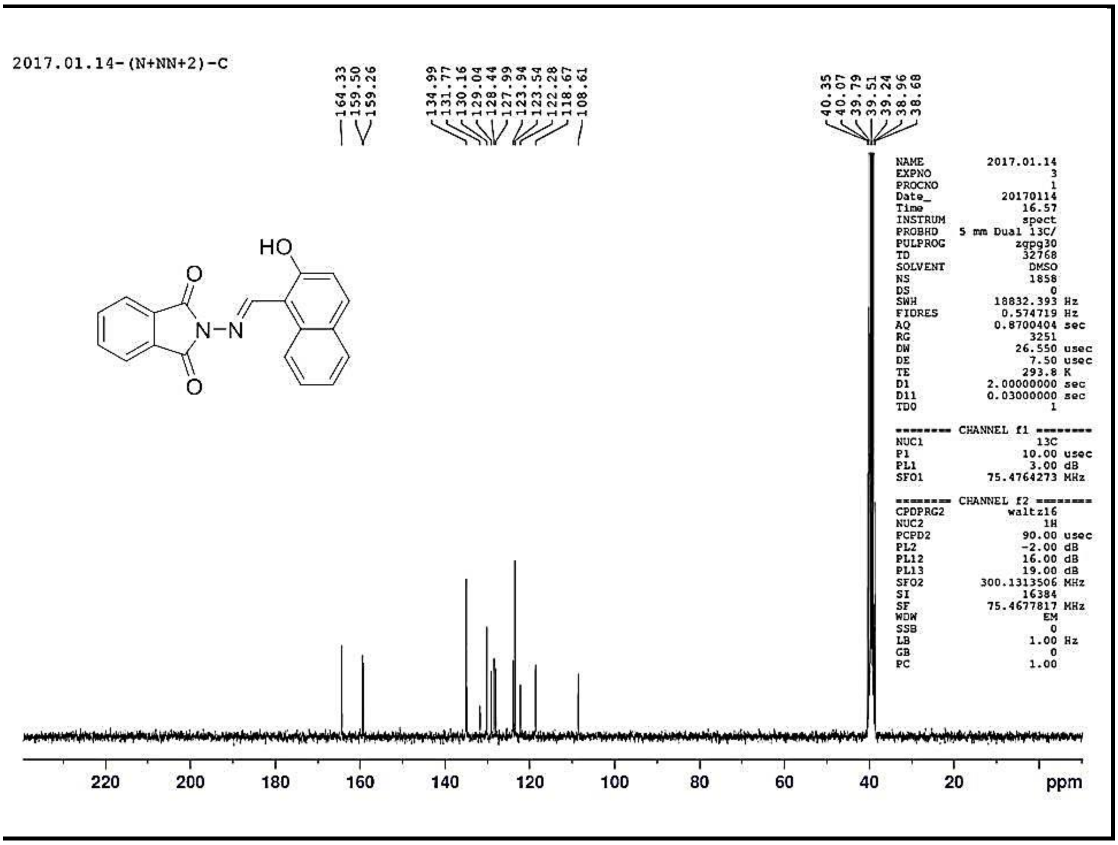

Fig. S2. ^{13}C NMR (DMSO-d_6) spectra for probe **L**

```
LIST: hei1143_no3(nn)-c1            24-Jan-17 REG   : 03:48.1     #9
Samp:                                         Start : 20:19:02   2065
Mode: EI +VE +LMR  ESCAN (EXP) UP HR NRM
Oper:                                         Inlet :
Limt: (   0)
    : (1391) C100.H100.N3.O3
Peak: 1000.00 mmu   R+D: -2.0 > 60.0
Data: +/777>1049  (CMASS : converted |CMASS : converted |CMASS : conv

              10587                  (mmu)
     Mass  Intensity     %RA   Flags Delta  R+D  Composition
316.0839    1058703   100.00   #       0.9  15.0  C19.H12.N2.O3
```

Fig. S3. High-resolution mass spectrometry data for probe **L**

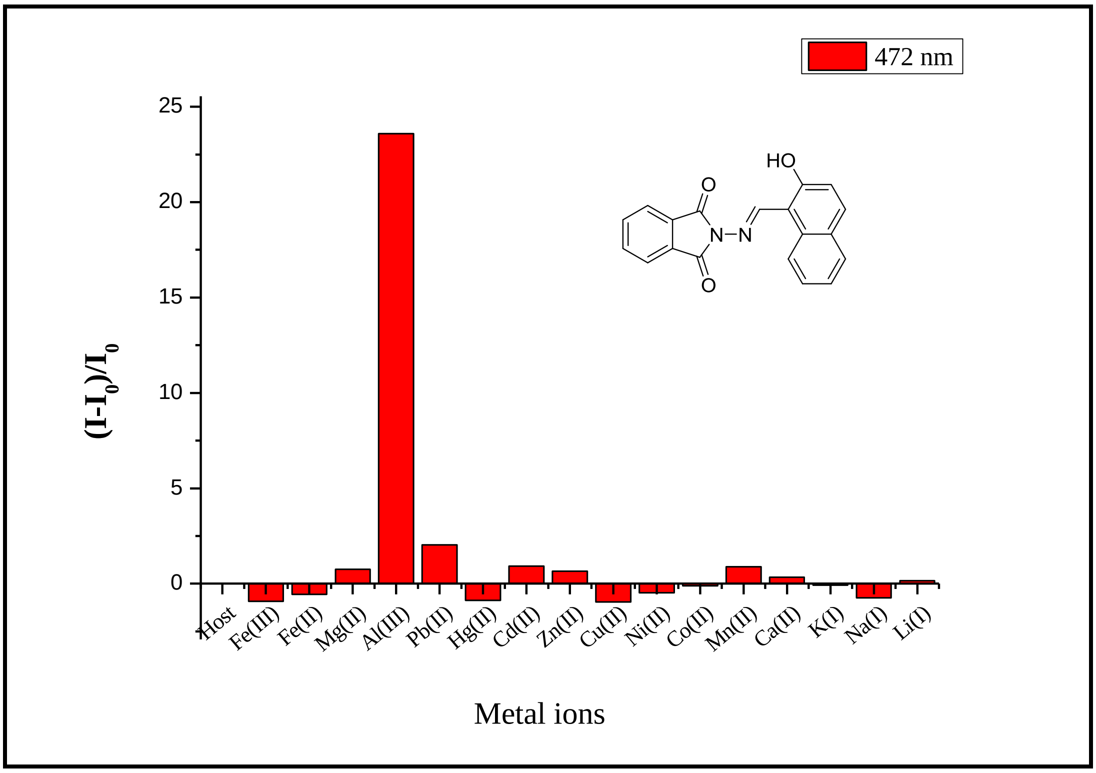

Fig. S4. Fluorescence intensity at 536 nm ($\lambda_{ex.}$ = 320 nm) for probe **L** (40 μM) in the presence of 5 equiv. of various cations in DMSO-H_2O (1:9 v/v).

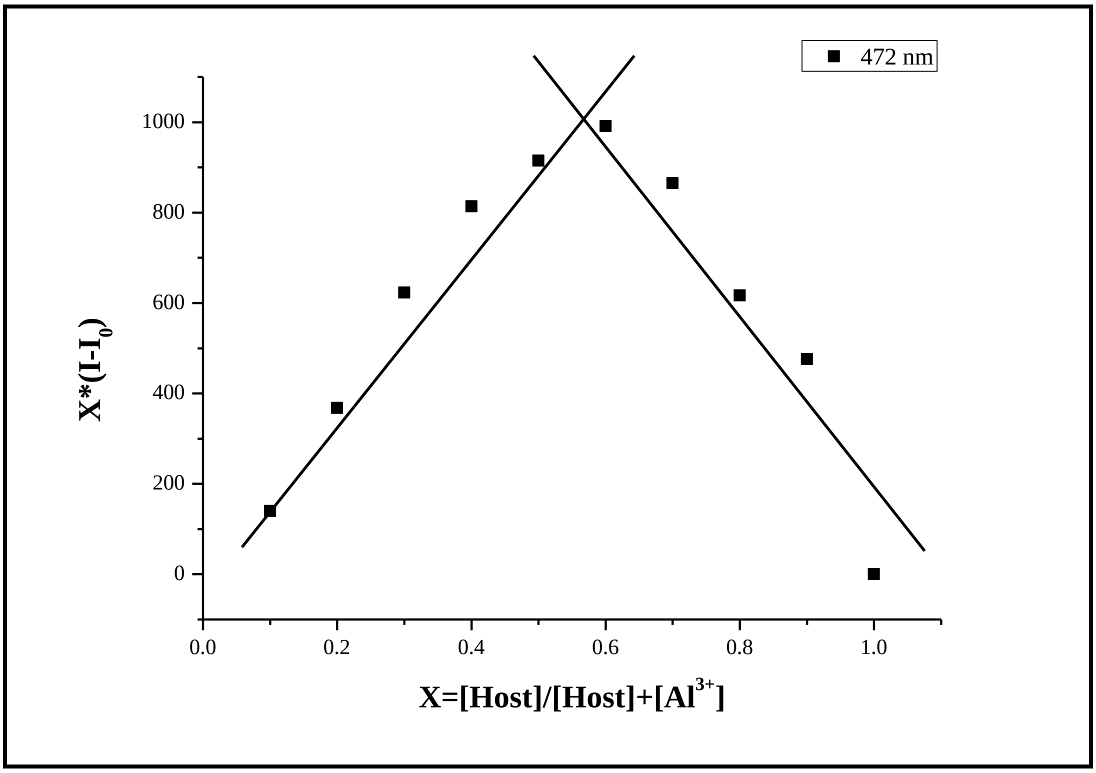

Fig. S5. Job plot of probe **L** and Al^{3+}

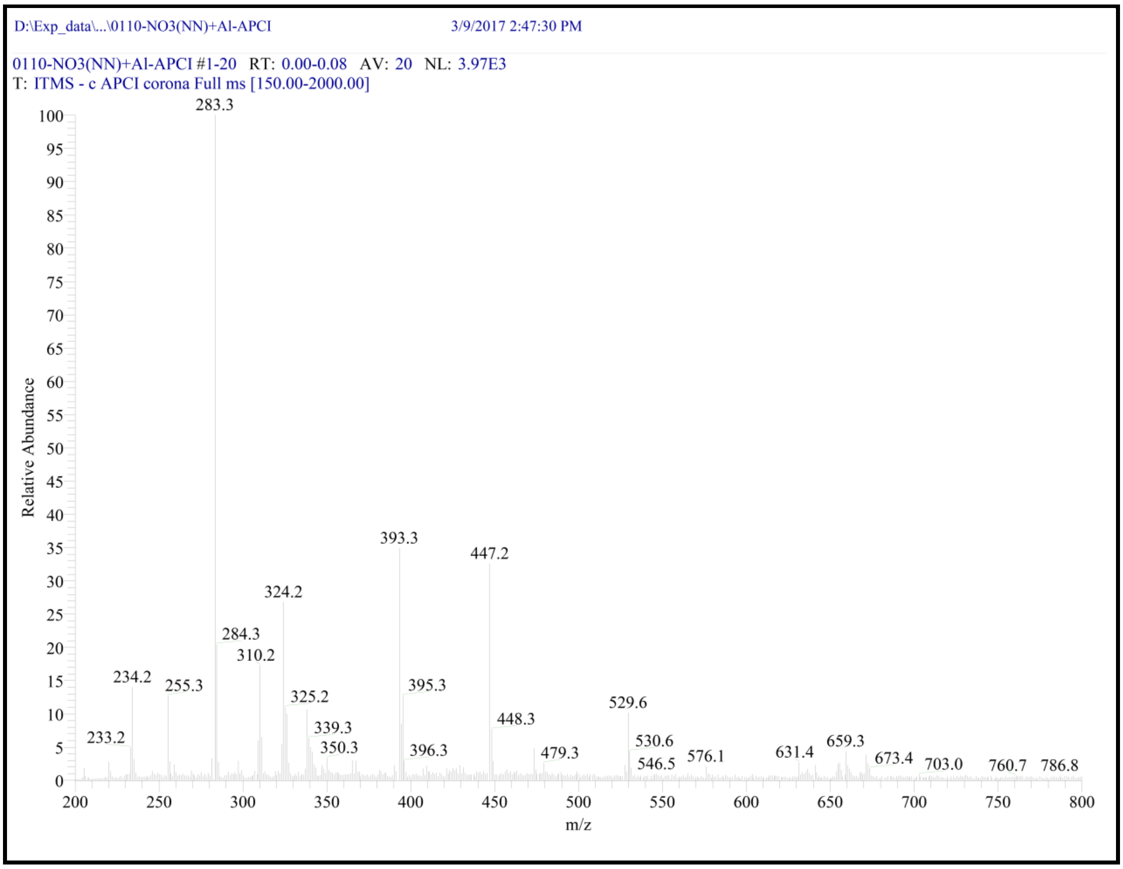

Fig.S6. ESI mass spectra [(probe **L** + Al^{3+} + DMSO + ClO_4^-)+$2Na^+$]

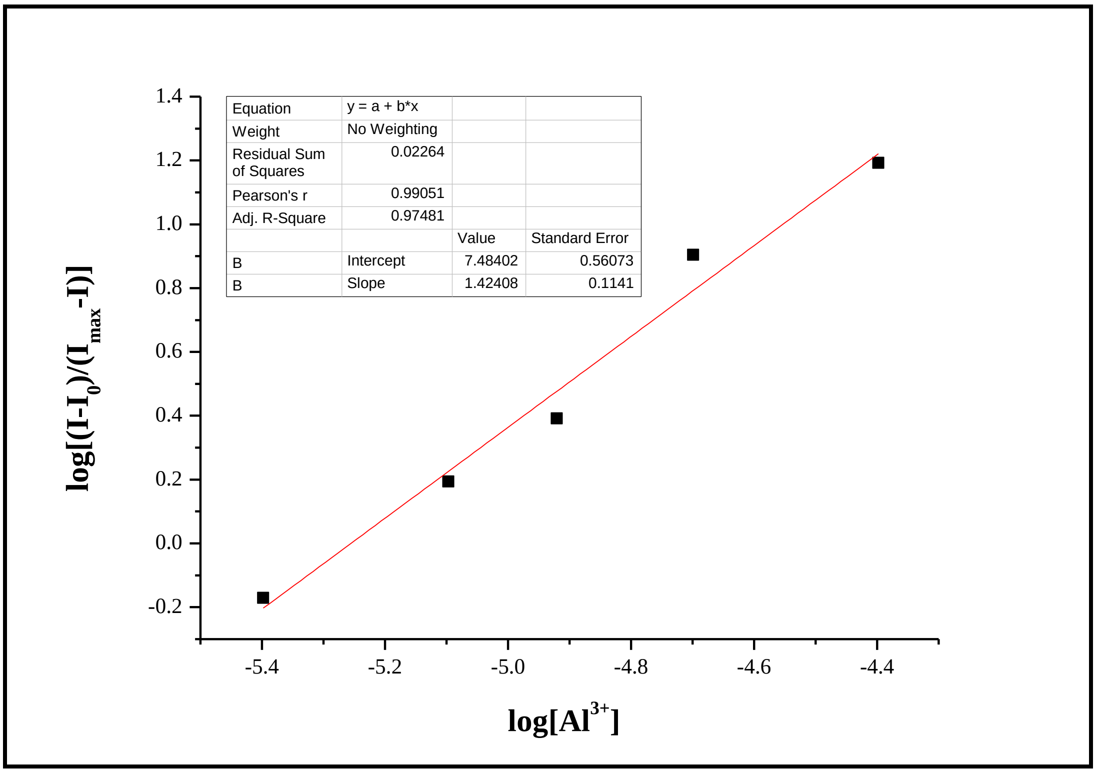

Fig. S7. Hill plot

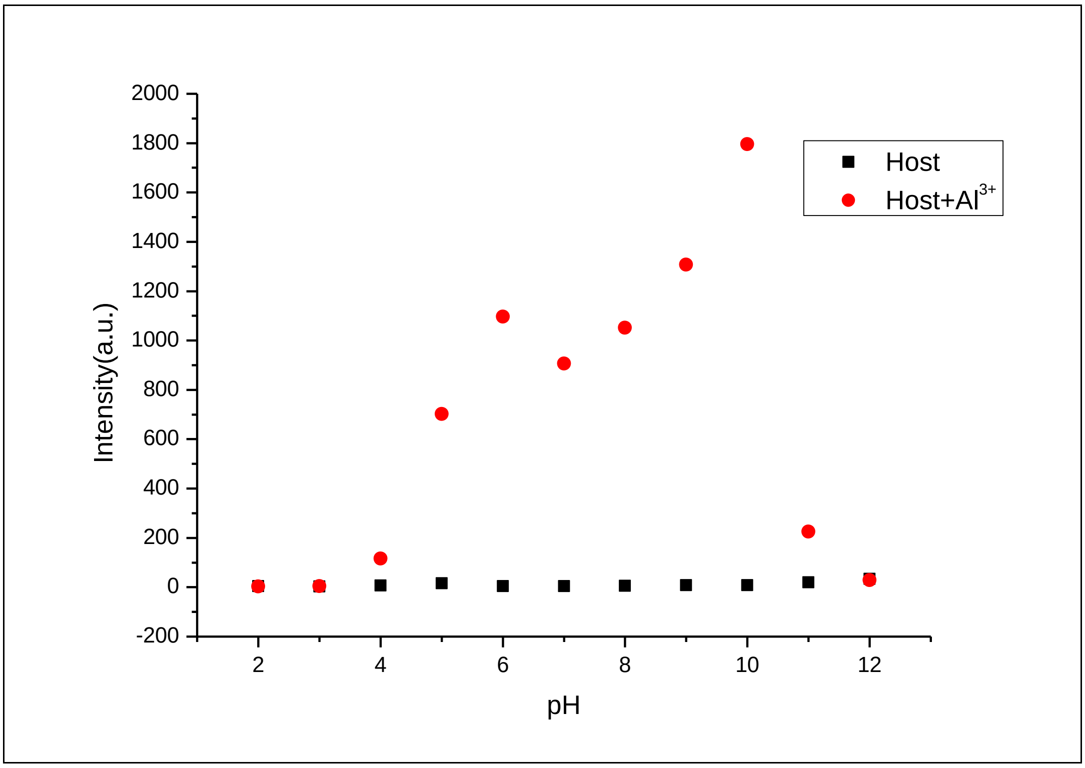

Fig. S8 Fluorescence emission intensity for probe **L** (40 μM) in the presence and absence of Al^{3+} (5 equiv) at different pH.

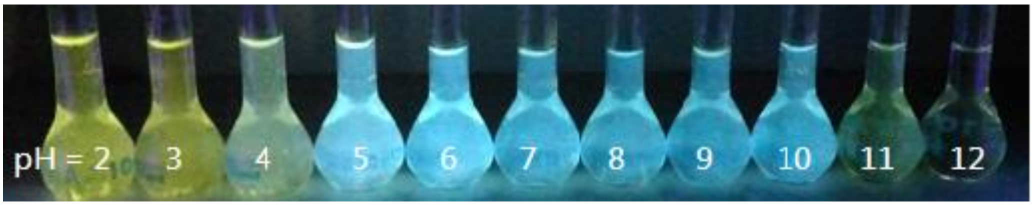

Fig. S9. Color changes by UV light for probe **L** with the addition of 5 equiv. of Al^{3+} at different pH value.
